# Establishing Covid-19 Research in UK Care Homes – Infrastructure Challenges for Trial Design

**DOI:** 10.1093/eurpub/ckac129.751

**Published:** 2022-10-25

**Authors:** P Leighton, K Sprange, K Robinson, C Rick

**Affiliations:** University of Nottingham, School of Medicine, Nottingham, UK

## Abstract

**Background:**

The Covid-19 pandemic brought into sharp relief the role that long-term care facilities play in health and social care of an aging population. It also cast a spotlight upon the need for high-quality research to assess the effectiveness of any care home interventions. The Prophylactic Therapies in Care Homes (PROTECT-CH) trial was one such study (funded by the UK National Institute for Health and Care Research). PROTECT-CH was designed to collect data in 200 care homes (approximately 6,400 residents), and whilst the changing epidemiology of Covid-19 in the UK (due to vaccination take-up) made this unfeasible other insights were gained about establishing large-scale research in care homes.

**Methods:**

An iterative process evaluation of the set-up phase of a large, platform trial testing prophylactic measures in long-term care facilities. Including a documentary review of the PROTECT-CH working groups and an online survey of working group members.

**Results:**

Documents were reviewed from 24 working groups, which in a hub and spoke model represented the PROTECT-CH trial infrastructure; representative of 20 of these groups completed an online survey about their organisation and working. Data demonstrated the number and organisation of individuals required to set up a large-scale care-home trial - 91 individuals representing a mix of academic, clinical, and methodological contributions from 25 organisations. Data demonstrated working groups specific to care home research, and activities designed to address the specific challenges of researching in care homes. PROTECT-CH produced dedicated training materials and reporting templates for care home research. PROTECT-CH established novel mechanisms for prescribing and clinical oversight in care home research.

**Conclusions:**

PROTECT-CH has highlighted the complexity of establishing large, scale RCT research in long-term care facilities. It has produced resources which might be of use in subsequent care home research.

**Key messages:**

• Infrastructure is required to support high quality research in long-term care facilities.

• RCTs in long-term care facilities pose specific challenged to researchers.

